# Comparative evaluation of uncertainty estimation and decomposition methods on liver segmentation

**DOI:** 10.1007/s11548-023-03001-1

**Published:** 2023-08-16

**Authors:** Vanja Sophie Cangalovic, Felix Thielke, Hans Meine

**Affiliations:** 1 Department of Computer Science, University of Bremen, Bremen, Germany; 2 Fraunhofer Institute for Digital Medicine MEVIS, Max-von-Laue-Str. 2, 28359 Bremen, Germany

**Keywords:** Image segmentation, Uncertainty, Bayesian neural networks

## Abstract

****Purpose**:**

Deep neural networks need to be able to indicate error likelihood via reliable estimates of their predictive uncertainty when used in high-risk scenarios, such as medical decision support. This work contributes a systematic overview of state-of-the-art approaches for decomposing predictive uncertainty into aleatoric and epistemic components, and a comprehensive comparison for Bayesian neural networks (BNNs) between mutual information decomposition and the explicit modelling of both uncertainty types via an additional loss-attenuating neuron.

****Methods**:**

Experiments are performed in the context of liver segmentation in CT scans. The quality of the uncertainty decomposition in the resulting uncertainty maps is qualitatively evaluated, and quantitative behaviour of decomposed uncertainties is systematically compared for different experiment settings with varying training set sizes, label noise, and distribution shifts.

****Results**:**

Our results show the mutual information decomposition to robustly yield meaningful aleatoric and epistemic uncertainty estimates, while the activation of the loss-attenuating neuron appears noisier with non-trivial convergence properties. We found that the addition of a heteroscedastic neuron does not significantly improve segmentation performance or calibration, while slightly improving the quality of uncertainty estimates.

****Conclusions**:**

Mutual information decomposition is simple to implement, has mathematically pleasing properties, and yields meaningful uncertainty estimates that behave as expected under controlled changes to our data set. The additional extension of BNNs with loss-attenuating neurons provides no improvement in terms of segmentation performance or calibration in our setting, but marginal benefits regarding the quality of decomposed uncertainties.

## Introduction

Segmentation is a cornerstone of automated medical image analysis. In this area of research, deep neural networks have pushed the limits and made even difficult applications feasible. However, many approaches have been shown to give overconfident predictions or to fail silently. At the same time, even humans cannot unambiguously decide for every single point in a medical image to which structure it belongs. This might be due to motion (e. g. breathing or cardiac motion), low contrast, noise, partial volume effects, or semantic ambiguities. Finally, by definition, patients do not only show *normal* anatomy, but medical imaging must cope with exceptions.

Most medical applications come with various degrees of risks and require robust and trustworthy algorithms, which motivates the search for reliable models that provide well-calibrated *uncertainty estimates*. This may help to prevent bad decisions based on erroneous results and might more generally increase trust in AI models. Finally, development itself can also benefit from uncertainty estimates, when active learning is used to focus the costly annotation on samples that most benefit the trained models.

Predictive uncertainty can be subdivided into two subtypes: *Aleatoric uncertainty* captures the noise or stochasticity inherent to the underlying process which generates the training data. *Epistemic uncertainty* represents the uncertainty of the model itself. The former is irreducible, while the latter can be explained away, e.g. by training on more data. This distinction increases the interpretability of uncertainty estimates, which is particularly desirable for human-in-the-loop scenarios. Moreover, uncertainty sampling in active learning can profit from high-quality epistemic estimates [1], while aleatoric uncertainty can be used to automatically detect annotation inconsistencies [2].

This work firstly aims at providing an overview of state-of-the-art uncertainty decomposition approaches with a focus on BNNs and loss attenuation. We then investigate how the quality of uncertainty decomposition compares between a plain BNN with mutual information measures and the combination of a BNN with an additional loss-attenuating neuron. In order to evaluate the quality of the decomposed uncertainties, we conduct experiments with varying training set sizes, artificial label noise, and distribution shifts.

## Related work

Probabilistic classifier neural networks (NNs) output categorical predictive distributions, which can be interpreted as capturing learnt aleatoric uncertainty, while measuring epistemic uncertainty would theoretically require a confidence estimate for the output probabilities [3]. However, the maximum softmax score has been shown to be a viable baseline for detecting misclassified as well as out-of-distribution (OOD) samples [4]. Cross-entropy loss—a proper scoring rule—promotes calibrated predictions, whereas Dice loss improves segmentation performance at the expense of reliability [5, 6]. A popular and successful approach to post-hoc calibrate NNs is to optimise a scalar temperature *T* by which all logits are scaled before the softmax operation [5].

### BNNs and MC dropout

In order to model epistemic, or model, uncertainty, network weights might be modelled as distributions $p(w \mid \mathcal {D})$. Bayesian inference then computes the posterior probability distribution over the weights *w* given a data set $\mathcal {D}$ where the likelihood $p(\mathcal {D} \mid w)$ quantifies how well the observed data can be explained.

The final posterior distribution allows to make predictions via Bayesian model averaging. Since marginalising over all possible weights is intractable, the predictive distribution is usually approximated via MC integration.

The same intractability arises during training. A popular and straightforward approach for approximating the posterior $p(w \mid \mathcal {D})$ is MC dropout [7], where multiple forward passes are interpreted as samples of the predictive distribution. MC dropout retains the computational efficiency of NNs at training time without architecture modifications and is thus a popular choice for approximating BNNs [8–10].

Given the predictive distribution, various measures for computing the model’s uncertainty have been proposed [11–13], such as the predictive entropy, the predictive variance, or the mutual information (MI) between the weights and the prediction. Nair et al [14] compare the performance of these three measures as well as the heteroscedastic uncertainty neuron (cf."Learnt loss attenuation" Section) for lesion segmentation on MRI sequences and found no differences apart from predictive variance estimates having lower magnitude.

### Learnt loss attenuation

While epistemic uncertainty can be modelled via weight uncertainty, aleatoric uncertainty can be implicitly learnt during training. If the model is able to gauge the input-specific amount of aleatoric uncertainty, it benefits from attenuating the loss for label-noisy samples.

DeVries and Taylor [15], for instance, propose to learn an additional confidence estimate regulating the amount of ground truth hints provided to the model at training time. Meanwhile, Thulasidasan et al [2] incorporate a learnt abstention class alongside the prediction, whose score lowers the loss directly and reliably indicates artificial label noise.

Analogously to regression uncertainty, Kendall and Gal [12] capture a classifier’s uncertainty via additional heteroscedastic uncertainty neurons (HUNs). The output of these neurons controls loss attenuation by placing Gaussian noise over the logits, resulting in a final model prediction $\textbf{p} = \textbf{z} + \mathbf {\sigma } * \epsilon \,$ based on the logit vector $\textbf{z}$ and a diagonal covariance matrix $\mathbf {\sigma }$ defined by the output of the HUN, $\epsilon \sim \mathbb {N}(0,\mathbb {I})$.

Meanwhile, Neumann et al [16] implement a heteroscedastic neuron outputting a confidence score $\alpha$ that scales the logits before the softmax operation. This approach has been termed “relaxed softmax” and can be interpreted as learnt heteroscedastic logit smoothing; we thus refer to the additional neuron as heteroscedastic logit smoothing neuron (HLSN) in the following.

The model’s confidence can also be used to smooth the targets, instead of the logits. McKinley et al [17] have shown label smoothing to improve classifier calibration and to also be directly learnable by the model.

### Decomposition of uncertainties

#### Kendall and Gal

Kendall and Gal [12] decompose a model’s predictive uncertainty into aleatoric and epistemic components by combining a BNN with additional heteroscedastic uncertainty neurons, as described in Sect.  "Learnt loss attenuation". Aleatoric uncertainty as seen in the training data lends itself to being captured by loss attenuation. Their setup jointly models both uncertainty types by explicitly using two mechanisms inside a single model. The authors found that modelling epistemic, aleatoric, as well as overall uncertainty improves segmentation performance over a vanilla NN baseline. The authors have also shown that for varying training set sizes and OOD inference the decomposed uncertainties quantitatively align with their definitions: Aleatoric uncertainty highlights segmentation boundaries, while epistemic uncertainty shows up in visually challenging pixels and instances of rare classes.

### Variance decomposition

Decomposing the predictive distribution of a BNN simplifies the architecture and has the theoretical advantage that the relationship between aleatoric and epistemic uncertainty can be modelled. Kwon et al [18] derive both aleatoric and epistemic uncertainty estimates from the variance of the predictive distribution: ${\text {Var}}_{p(y \mid x)}(y) = \mathbb {E}_{p(y \mid x)}\{y^{\otimes 2}\} - \mathbb {E}_{p(y \mid x)}\{y\}^{\otimes 2}$ with $y^{\otimes 2} = yy^T$. The aleatoric term computes the variance of individual predictions, while epistemic uncertainty corresponds to the variability of the network weights.

The authors compare their approach to that of Kendall and Gal [12], employing the heteroscedastic neuron activation as aleatoric estimate, for a stroke lesion segmentation task. Their resulting uncertainty maps for Kendall and Gal’s approach show next to no aleatoric uncertainty, and epistemic uncertainty only appears diffusely in the background. Meanwhile, their proposed aleatoric and epistemic estimates highlight segmentation borders and incorrectly segmented regions, the latter having lower magnitude overall. Furthermore, the authors find increased epistemic uncertainty in models trained on smaller data subsets, while aleatoric uncertainty stays constant.

#### Mutual information decomposition

Similar to the variance-based approach, ${\text {MI}}[y_i, W \mid x_i, \mathcal {D}] \approx {\text {H}}[y_i \mid x_i, \mathcal {D}] - \mathbb {E}[{\text {H}}[y_i \mid x_i,W]]$ gives the reduction in uncertainty for the weights *W* given a sample $x_i$ and the target label $y_i$. The predictive entropy (minuend) represents the overall uncertainty, and the subtrahend eliminates weight uncertainty by conditioning on *W*. In practice, this translates to computing the average entropy of the individual predictions, which corresponds to measuring aleatoric uncertainty. Thus, epistemic uncertainty is computed by subtracting aleatoric uncertainty from the predictive entropy [19]. In Mobiny et al [11], the resulting uncertainty maps for three different segmentation tasks show clear correlations between epistemic uncertainty and segmentation boundaries, class label frequency, and visual ambiguity of objects.

## Materials and methods

We employ a subset of the LiTS data set [20], a collection of abdominal CT scans with annotated liver and tumour tissue collected from seven source institutions. Since this work’s intent is not to participate in that challenge, we optimise for faster trainings, resampling to a uniform voxel size of $(2.5\,\textrm{mm})^3$ and transversal view direction and casting tumour annotations to liver, binarising the labels. Out of 131 annotated CT scans, we exclude 59 with questionable annotations, then use 32 volumes as default training set (“LiTS-32” below), another 33 cases as test set (LiTS-test), and 7 cases for validation.

### Model architecture and training

We train and evaluate two architectures: a plain BNN, approximated via MC dropout, and one extended with an HLSN. In both cases, the base architecture is a fully convolutional, 5-level, 3D, anisotropic u-net [21], a variant of the very popular u-net architecture. We employ decoder-only dropout with rate 0.2 [12] and draw 20 samples per prediction. Training and inference are performed on image patches of size $236 \times 236 \times 156$ voxels with “valid” mode convolutions. During training, patches containing foreground were sampled more often in order to stabilise the training, increasing their frequency from 33 to 80%. Training employs the Adam optimiser, a batch size of 2, and is stopped once the validation Jaccard performance has not improved for 40 epochs. We optimise the cross-entropy loss as usual for uncertainty quantification [11, 12, 16, 18], but also examine the influence of Dice loss on the decomposed uncertainties.

### Heteroscedastic logit smoothing

Heteroscedastic uncertainty neurons [12] induce loss attenuation by sampling Gaussians through the softmax, which asymmetrically squashes the sampled logits, so that a large variance brings the resulting prediction closer to a uniform distribution. The more recently published HLSNs [16] achieve a similar smoothing of the output scores by explicitly scaling the logits with a learnt $\alpha$. We analysed the two approaches mathematically and experimentally and conclude that they perform similarly. Hence, we combine BNNs with HLSNs in this work, which do not require sampling. A sigmoid activation for $\alpha$ is employed to allow only for the (smoothing) downscaling of logits.

Reminiscent of custom training regimes employed for other loss attenuation approaches, we let models enter training with high confidence by initialising incoming weights to $\textbf{w} \sim \mathcal {N}(1,0.6)$. In preliminary experiments, this lets the HLSNs converge more robustly towards meaningful uncertainty estimates.

#### Aleatoric uncertainty from loss-attenuating neurons

Intuitively, the activation of loss-attenuating neurons constitutes an uncertainty estimate, since the model learns to produce high activations for difficult, ambiguous samples. Notably, many works reproducing Kendall and Gal’s HUN use this straightforward interpretation [14, 15, 18]. However, the raw neuron activation does not correspond to any well-defined measure, and the degree of loss attenuation it induces also depends on the logits’ magnitudes. Moreover, the logits themselves inherently contain aleatoric uncertainty. Therefore, one should not directly interpret the uncertainty neuron’s activation, so we primarily present aleatoric uncertainty as measured via the average entropy component in MI, which is based on the smoothed output distribution, but will also present the HLSN activation for comparison.

**Fig. 1:**
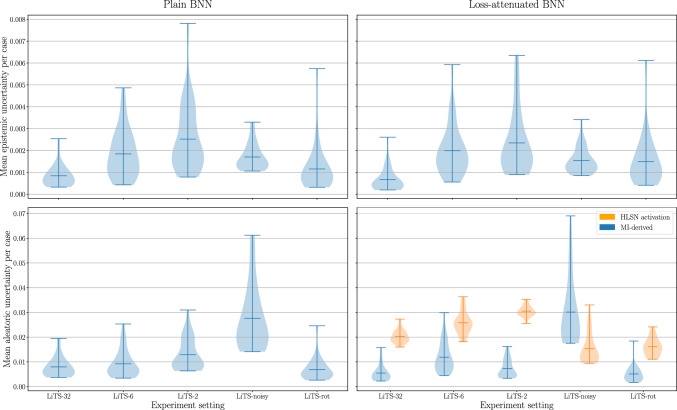
Mean per-case uncertainty estimates of plain and loss-attenuated BNN

### Experiments

In every experimental setting, we train three models and average the resulting per-case uncertainties. We assess differences via Wilcoxon signed-rank testing.

### Varying training set size

Reducing the training data constrains a model’s knowledge and should increase epistemic uncertainty, while aleatoric uncertainty is expected to stay the same, since the amount of label noise does not change. The different training sets used are the full LiTS-32 and random subsets of six (LiTS-6) and of two cases (LiTS-2), with LiTS-2_i_ $\subset$ LiTS-6_i_ $\subset$ LiTS-32 for $i \in {1,2,3}$. Different subsets *i* are employed for each run to minimise the influence of subset-specific patterns.

### Artificial label noise

We also compare models trained with LiTS-32 and models trained on a variant LiTS-noisy derived by adding label noise to one half of the label masks. This artificial noise is created by dilating the liver masks with a $7\times 7 \times 1$ kernel, mimicking annotations produced via different annotation regimes. Models trained on LiTS-noisy are expected to produce higher aleatoric uncertainty around the border of the liver, while epistemic uncertainty should stay similar.

### Out-of-distribution (OOD) inference

Ideally, medical image segmentation models should indicate whether a given sample is OOD via raised epistemic uncertainty. In a clinical setting, varying imaging protocols, patient characteristics, or pathologies may cause images to diverge from those seen during training. Hence, all models trained on LiTS-32 are tested on 33 test cases that were rotated 180 degrees (LiTS-rot). In addition, we perform tests on a CT scan taken from an internal data set, which displays a severe form of ascites not encountered to this extent in LiTS.

## Results and discussion

**Fig. 2:**
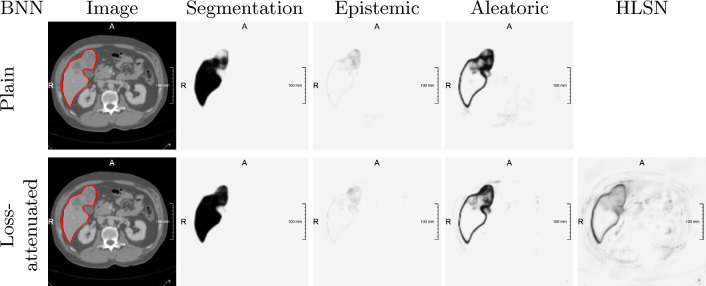
Input with ground-truth liver mask, segmentation, and uncertainty maps for models trained on LiTS-32

Before we present the quantitative evaluation of our experiments, we briefly describe qualitative observations. As shown in Fig. 2, aleatoric estimates consistently highlight segmentation boundaries. Epistemic uncertainty frequently appears similar, though less focal and only partially highlighting segmentation borders, as well as having vastly lower magnitude. Our binary classification setting might explain the latter observation, since neither rare classes nor visually particularly difficult pixels are common. These findings are consistent with other works [11, 12, 14, 18].

Moreover, aleatoric uncertainty derived via MI (from both architectures) appears more crisp than the HLSN activation, which sometimes also dimly highlights the borders of background structures. Similar behaviour has been reported for Kendall and Gal’s HUN activation [14, 18].

On models trained with Dice loss, we observe reversed results; epistemic estimates appear at class boundaries while aleatoric estimates are reduced to a faint border, which aligns well with Dice loss’ known tendency to promote overconfident predictions [6]. We also find that loss attenuation neither rectifies this miscalibration nor restores uncertainty decomposability.

For all experiment settings, segmentation performance (as measured via Dice index) and model calibration (as measured via NLL) do not significantly differ between plain and loss-attenuated BNNs.

### Varying training set size

Training on fewer data results in significantly higher epistemic uncertainty for both model architectures (*p*-values $\ll 0.01$), as shown in Fig. 1. This increase in epistemic uncertainty is statistically significantly higher for loss-attenuated BNNs than for plain BNNs on LiTS-6 (*p*-values $\ll 0.01$), and comparable on LiTS-2. Epistemic estimates occur at segmentation boundaries, as well as occasionally highlighting the spleen or kidney, demonstrating the model’s lack of knowledge regarding features that differentiate the liver from organs with similar radiodensity. The overall behaviour of epistemic uncertainty thus conforms to its definition.

Aleatoric uncertainty estimates of both architectures marginally increase when training on smaller data sets, as shown in Fig. 1. This can be explained by aleatoric uncertainty bordering false negative liver lesions, whose number increases with decreasing training set sizes.

### Artificial label noise

**Fig. 3:**
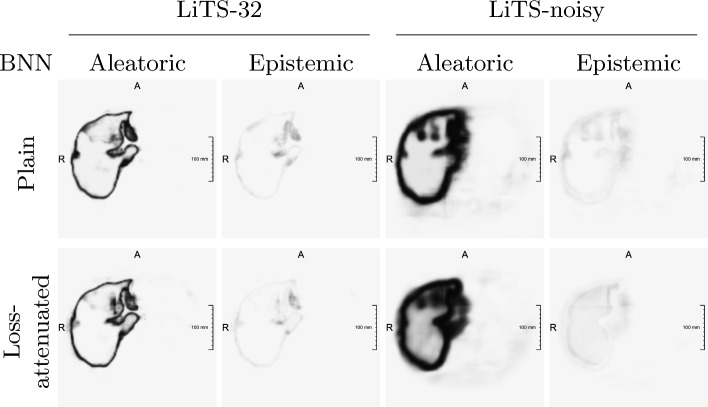
Uncertainty maps for models trained on LiTS-32 vs. LiTS-noisy

In line with their definition, introducing artificial label noise substantially increases aleatoric uncertainty estimates for both architectures when derived via MI (*p*-values $< 0.0001$), as shown in Fig. 1 (LiTS-noisy vs. LiTS-32). The increase in aleatoric uncertainty is statistically significantly higher for loss-attenuated BNNs than for plain BNNs (*p*-values $\ll 0.01$). Figure 3 reveals broadened estimates around the segmented region. The magnitude of the HLSN activation, on the other hand, varies visibly across the three trained models. This lack of robustness results in a mean decrease of aleatoric uncertainty, as shown in Fig. 1.

Epistemic uncertainty is quantitatively slightly increased, blurred and outlines both the undilated liver and its broadened outline. Considering that the models were essentially trained with two segmentation variants, observing epistemic uncertainty around both learnt contours appears reasonable.

### OOD inference

On LiTS-rot, aleatoric and epistemic uncertainty for plain and loss-attenuated BNNs both highlight regions of the segmented liver, with aleatoric uncertainty also outlining the confidently segmented spleen. Figure 1 shows that mean aleatoric uncertainty of both model architectures is slightly decreased while epistemic uncertainty is considerably increased ($p < 0.05$). Since LiTS-rot and LiTS-test contain the same amount of label noise by construction, the decrease in aleatoric uncertainty has to be explained by the model’s failed segmentation. The increase in epistemic uncertainty is statistically significantly higher for loss-attenuated BNNs than for plain BNNs (*p*-values $< 0.01$).

**Fig. 4:**
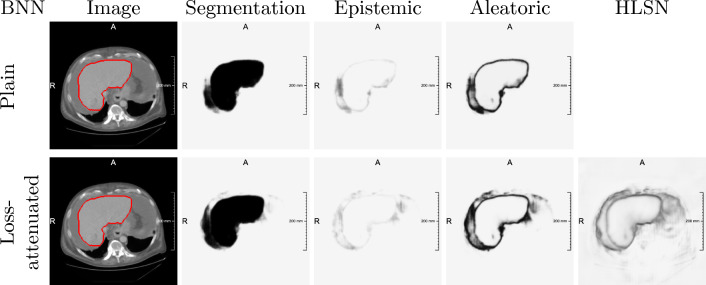
Uncertainty maps derived from plain and loss-attenuated BNN models trained on LiTS-32 and inferring on an OOD case with ascites

Both architectures perform meaningful liver segmentations on the ascites case, only mistaking parts of intraperitoneal fluid as liver. This aligns with the case’s arguably smaller shift in distribution. Figure 4 shows that uncertainty estimates not only highlight the boundary of the segmented liver region, but also the false positive areas, successfully indicating the distribution shift.

### Aleatoric uncertainty from loss-attenuating neurons

The observed qualitative differences between the two aleatoric uncertainty estimates derived from loss-attenuated BNNs are in line with prior work, in which the HUN activation was found to highlight the borders of the segmented objects as well as those of other structures [14]. Moreover, the quantitative evaluation of the behaviour of both aleatoric uncertainty measures shows that MI-based estimates correspond precisely to their definition while the HLSN activation is less cleanly interpretable. Furthermore, we found the logits of loss-attenuated BNNs to convey a visible amount of uncertainty, suggesting that the calibration lever introduced by the HLSN is used *in addition to* and not *as a replacement for* logit-inherent uncertainty. These findings corroborate the theoretical discussion in Sect. "Materials and Methods" on “aleatoric uncertainty from loss-attenuating neurons”, motivating the use of comprehensive MI-based measures.

## Conclusion and outlook

This work thoroughly compares the explicitly separate modelling of epistemic and aleatoric uncertainty in a BNN with a loss-attenuating neuron and the decomposition of the predictive uncertainty of a BNN via MI on the task of liver segmentation in CT. BNNs in this work are implemented via MC dropout and loss attenuation via heteroscedastic logit smoothing neurons. The results show that the overall behaviour of both aleatoric and epistemic uncertainties derived from both model architectures via MI is consistent with their respective definitions for varying training set sizes, label noise, and distribution shifts.

Meanwhile, we found the HLSN activation to be noisier and less robust in capturing uncertainty than aleatoric estimates derived via MI. This leads us to the conclusion that MI appears suited for uncertainty decomposition of BNNs with and without loss-attenuating neurons, the addition of which slightly improved the quality of decomposed uncertainties, but did not provide statistically significant benefits in our example context in terms of liver segmentation performance or reliability while requiring some architectural extension and careful initialisation.

In the future, we hope that our conclusions on decomposed uncertainty estimation can be confirmed on more diverse multi-class data sets.
